# Infantile osteomyelitis of the fourth rib causing the occurrence of a lung abscess

**DOI:** 10.1002/ccr3.2226

**Published:** 2019-05-28

**Authors:** Sayo Mori, Sagano Onoyama, Tomoko Takimoto, Yuya Morooka, Kenji Furuno

**Affiliations:** ^1^ Department of General Pediatrics & Interdisciplinary Medicine Fukuoka Children’s Hospital Fukuoka Japan; ^2^ Department of Pediatric Infection Disease Fukuoka Children’s Hospital Fukuoka Japan

**Keywords:** chest X‐ray, computed tomography, fever in infants, magnetic resonance imaging, *Staphylococcus aureus*

## Abstract

Infantile osteomyelitis of the rib is rare but can be complicated by intrapleural pyogenic lesions. Even if findings suggest another infection focus, osteomyelitis should be considered if there are changes on radiographs. In addition, it can be prevented by maintaining the dermal barrier function through skin care.

## 1 INTRODUCTION

Bacterial infections in infants under 3 months old tend to rapidly become exacerbated and cause a life‐threatening condition. Therefore, the early diagnosis and appropriate treatment are critical. However, it can be difficult to identify infection foci in young infants under 3 months old, as specific physical findings are not often observed. While hematogenous osteomyelitis is a serious disease, it is difficult to diagnose, because few specific symptoms and findings are observed in the early stages of the disease.^1^

We herein report the case of a 1‐month‐old infant who was eventually diagnosed with osteomyelitis of the rib while undergoing treatment for pneumonia following an initial diagnosis based on chest X‐ray.

## 2 CASE HISTORY

A 41‐day‐old male infant presented with a fever of 38.2°C and was admitted to our hospital. He also had a 2‐week history of irritability and a transient fever 5 days before admission.

**Figure 1:**
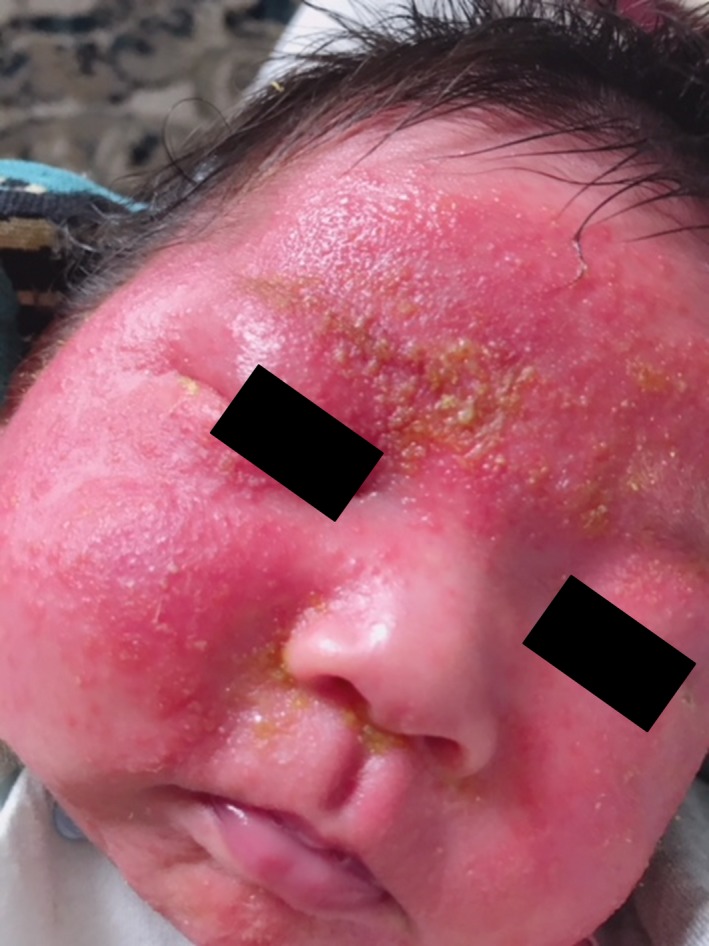
The face was covered with eczema and partially accompanied by exudate at presentation to the hospital

**Figure 2:**
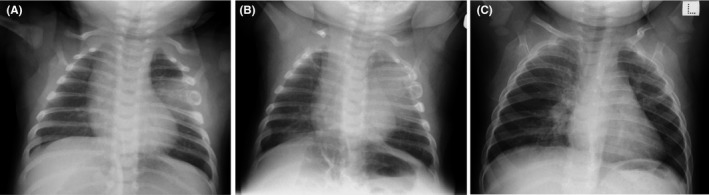
Chest X‐ray showing an area of reduced permeability in the left middle lung field and a bone lesion of the fourth rib on admission (A) and on the 8th day of hospitalization (B). One year later, neither bone changes nor lung lesions could be identified (C)

At the time of admission, his activity was poor, and he was irritable. The skin of the whole body was covered with eczema, and that of the face was accompanied by exudate (Figure 1). The vital signs were as follows: a heart rate of 167 bpm, respiratory rate of 60 breaths/min, SpO_2_ of 98% (room air), and body temperature of 39.5°C. Physical findings included a flat anterior fontanel, no decreased or left‐right difference in respiratory sounds, no deformation of the thorax, no abnormal movement of the limbs, and no sensations of cold in the peripheral limbs. A blood test showed a white blood cell count of 20 600/µL (neutrophils 53.9%, lymphocytes 33.4%) and C‐reactive protein of 8.18 mg/dL. The cerebrospinal fluid was clear and did not show an increase in the cell count or a decrease in glucose levels. Chest X‐ray showed an area of reduced permeability in the left middle lung field. The inside of the fourth rib appeared to be enlarged around the tuberosity for serratus anterior (Figure 2A). Based on chest X‐ray findings, he was considered to have pneumonia (enlargement of the rib was not considered pathological). Given his age, we commenced the administration of antibiotics with ampicillin (ABPC) at a dosage of 175 mg/kg/d and cefotaxime (CTX) at 175 mg/kg/d.

**Figure 3:**
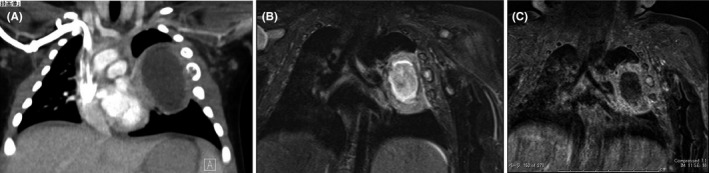
Computed tomography showing a lung abscess (4 × 5 × 3 cm). The fourth rib adjacent to the mass was irregularly enlarged, and the cortex had been destroyed (A). Fat‐suppressed T2‐weighted (B) and postcontrast fat‐suppressed T1‐weighted (C) magnetic resonance imaging showing hypersignal intensity in the irregularly enlarged fourth rib, suggesting osteomyelitis. No abnormal signals were seen in the third or fifth ribs

Progress was favorable. He showed rapid clinical improvement by the third day following the initiation of treatment. Since cultures of the urine, cerebrospinal fluid, and blood were all negative, we discontinued cefotaxime on the 7th day of hospitalization. Follow‐up X‐ray on the 8th day of hospitalization revealed a broadened area of permeability in the left lung field along with enlargement of the rib with the destruction of the cortex (Figure 2B). Therefore, contrast‐enhanced chest computed tomography (CT) was performed. A thick‐walled fluid‐filled cavity of 4 × 5 × 3 cm, which was considered to be a lung abscess, was observed in the left chest cavity. The fourth rib, which was attached to the mass, was irregularly swollen, and its cortex was ruptured (Figure 3A). Coronal fat suppression magnetic resonance imaging (MRI) demonstrated irregular swelling of the fourth rib and an internal area with an enhanced fat‐suppressed T2‐weighted signal. No abnormal signals or intensity was observed on the third and fifth ribs; however, they were also attached to the abscess (Figure 3B). Gram stain results of the abscess puncture fluid showed Gram‐positive cocci, thought to be *Staphylococcus aureus*. The results of culture indicated methicillin‐susceptible *S aureus* (MSSA). On day 10 of hospitalization, antibiotics were changed to cefazolin (CEZ) 200 mg/kg/d.

Following this treatment change, progress was favorable. After 4 weeks of the intravenous administration of CEZ for osteomyelitis, the medication was changed to the oral administration of cephalexin (CEX) 100 mg/kg/d. The patient was discharged having completed a total of 8 weeks of antibiotic therapy.

For a year following discharge, the patient had no relapse, and the changes in the rib mostly improved (Figure 2C). At the same time as these antimicrobial treatments were being applied, a dermatologist also treated the patient for the skin lesions using steroids and moisturizers.

## 3 DISCUSSION

We encountered a 1‐month‐old boy with infantile eczema who was diagnosed with MSSA‐induced osteomyelitis of the rib and a lung abscess during treatment for pneumonia. The patient visited our department with a fever and poor activity. Based on the findings from chest X‐ray, we initially diagnosed the patient with pneumonia and started the administration of antibiotics.

The incidence rate of pediatric hematogenous osteomyelitis is 1 in 500‐2300 in developing countries and 1 in 5000‐7700 in developed countries. The disease is more common in children than in adults; however, the proportion of cases with rib lesions is 0.6%. Osteomyelitis of flat bones in neonates is also rare.^1^ To our knowledge, there have been only two reports of osteomyelitis of the rib in immunocompetent neonates.^2^, ^3^ In the present case, chest X‐ray at the time of admission showed swelling of the fourth rib, which was in contact with an area with reduced permeability in the left middle lung field. However, we were unable to determine the causal relationship between the swelling in the rib and pyrexia. In addition, we failed to detect any local symptoms, such as swelling or redness.

Bacterial osteomyelitis has three pathways of infection: hematogenous infection, infection related to an adjacent pyogenic focus, or as a result of trauma. In the present case, it was suggested that MSSA entered the bloodstream through the inflamed skin and was hematogenously trapped in the rib bone marrow. It has been reported that infection tends to spread from osteomyelitis to the peripheral soft tissue and neighboring joints in newborns and young infants because the bone cortex and subperiosteal tissue are loose at that age. We also considered the present case to have been complicated with a lung abscess following osteomyelitis of the rib. If the lung abscess was the primary infectious focus, then this case could have been accompanied by pleural effusion, thoracic empyema, and other respiratory symptoms. In addition, the inflammation of the soft tissue of chest wall was observed at the same time as bone invasion. The present patient had neither a history of trauma nor scarring. Blood culture is positive in only 40% of patients with hematogenous osteomyelitis. Negative results of blood culture do not constitute a rationale for ruling out hematogenous osteomyelitis.

The patient was suffering from severe infantile eczema with effusion prior to the hospitalization and diagnosed with atopic dermatitis thereafter. The dermal barrier function of the patient was considered to be damaged. There have been many reports of cases of atopic dermatitis with bacteremia. Beneson et al stated that the skin of patients with atopic dermatitis provided an environment that facilitated the colonization, proliferation, and invasion of *S aureus*. Therefore, atopic dermatitis is a risk factor of *S aureus* bacteremia.^4^ Recently, it has been found that moisturizing the skin from an early stage of infancy improves the dermal barrier function.^5^ If the patient in our case had been provided adequate skin care, infection could have been prevented.

Another important point to note in the present case is that we commenced treatment for pneumonia at the time of admission. Contrast CT was performed due to poor improvement in chest X‐ray images, which led to a diagnosis of osteomyelitis of the rib. However, osteomyelitis should have been suspected based on the left‐right difference in the rib on chest X‐ray at time of admission. Signs of osteomyelitis in plain radiographs usually do not appear until 10‐14 days after the onset of symptoms.^6^ In many cases, the clinical symptoms of pediatric osteomyelitis are nonspecific and not accompanied by pyrexia.^1^ In the present case, changes in the rib were already observed at the time of admission. The onset of osteomyelitis may have occurred around the age of 26 days, when he became irritable. In addition, the estimated causal organism and treatment periods differ between osteomyelitis and pneumonia. The risk of delaying treatment for osteomyelitis patients may be significant, particularly for those with *S aureus*. For empiric parenteral treatment in young infants, an antistaphylococcal agent should be used with third or fourth generation cephalosporin. The duration of antibiotic treatment is debatable. It generally takes 4‐6 weeks or more to be treated.^7^ Thus, it is important to carefully observe bone changes on X‐rays, even if there are findings that suggest a focus of another disease.

## 4 CONCLUSION

The infant in our case suffered from a complication of osteomyelitis of the rib and a lung abscess caused by *S aureus* that invaded the skin due to a reduced barrier function, causing a hematogenous infection. While the prognosis of infantile eczema is generally favorable, damage to the dermal barrier function may be a risk factor for life‐threatening bacterial infection, particularly in newborn babies and those in early stages of infancy. Although the present case is a rare case, when evaluating skin care in infantile eczema and the cause of pyrexia, it is necessary to be alert for slight changes in the bone on X‐ray.

## CONFLICT OF INTEREST

None declared.

## AUTHOR CONTRIBUTION

SM and TT: were responsible for the initial draft of this manuscript. SO and KF: have reviewed this manuscript and made edits to the text. YM: contributed to write the case and identify the images. KF: is the named physician who is responsible for this patient's care. All the authors approved the final version for submission.

## CONSENT CONFIRMATION

Consent was obtained from the patient for publication of case details.
